# Psychiatric and Neurological Manifestations of Celiac Disease in Adults

**DOI:** 10.7759/cureus.35712

**Published:** 2023-03-03

**Authors:** Resheed Alkhiari

**Affiliations:** 1 Department of Medicine, Qassim University, Qassim, SAU

**Keywords:** gluten, auto immune, gastrointestinal, psychiatric manifestations, celiac disease

## Abstract

Celiac disease (CD), a chronic inflammatory disorder of the intestines, affects 0.7% to 1.4% of the world's population. CD causes diarrhea, abdominal discomfort, bloating, flatulence, and, in rare cases, constipation in the digestive tract. Since the identification of gluten as the disease-causing antigen, CD patients have been treated with a gluten-free diet, which is advantageous but has limitations for certain patient groups. CD is associated with mood disorders, such as manic-depressive disease, schizophrenia, and bipolar disorder, as well as other disorders such as depression and anxiety. The relationship between CD and psychological issues is not entirely understood. Here, we look at the most recent psychiatric data as they pertain to CD, as well as the relevant psychiatric manifestations that have been associated with this condition. Clinicians should examine mental health factors when a CD diagnosis is established. More research is needed to understand the pathophysiology of CD's psychiatric manifestations.

## Introduction and background

Celiac disease (CD) is a common chronic inflammatory illness caused by gluten consumption in genetically predisposed individuals and characterized by gluten-induced immune-mediated enteropathy [1]. The primary clinical manifestation is an autoimmune enteropathy linked to a particular set of circulating autoantibodies and a particular haplotype of the human leukocyte antigen (HLA-DQ2 or HLA-DQ8) [2]. Coeliac enteropathy is caused by a complicated immunological response to gluten proteins, involving both adaptive and innate mechanisms. In the general population, prevalence ranges from 0.5% to 2%, with an average of approximately 1% [3].

The clinical signs of CD are exceedingly heterogeneous, ranging from malabsorption to extraintestinal manifestations to silent states. As a result, the majority of people with CD are undiagnosed, misdiagnosed, or identified with a significant delay [4]. Failure to thrive, short stature, delayed puberty, exhaustion, and loss of weight are all possible symptoms of CD. It's crucial to keep in mind, though, that 10% of CD patients are obese. Gastric symptoms that might go along with CD include nausea, electrolyte imbalance, cramping, bloating, flatulence, and diarrhea. Non-classical CD symptoms include iron deficiency anemia, increased transaminases, constipation, ataxia, lethargy, osteoporosis, thrombotic events, and dyspepsia [5-7].

CD has the same frequency of extraintestinal symptoms in both children and adults. Anemia, exhaustion, and headache/psychiatric disorders were the most common in adults while short stature, fatigue, and headache were most common in children. When compared to adults, symptoms improved more quickly in children who followed a gluten-free diet (GFD). Children of small stature who are unresponsive need to be checked for co-occurring conditions [8].

Gluten sensitivity (GS) or non-celiac gluten sensitivity (NCGS) is a form of gluten-related disorder that is characterized by a set of symptoms resembling celiac disease without immunological involvement. The classic symptoms of GS are similar to irritable bowel syndrome like abdominal pain, altered bowel habits, and bloating, and they usually occur after gluten ingestion. GS can be challenging, and diagnosis is usually made based on suspicion of clinical symptoms, exclusion of celiac disease, and response to a gluten-free diet [9].

CD and gluten sensitivity (GS) can both cause neurological and psychiatric complications; however, patients with gluten sensitivity may experience more extraintestinal symptoms than CD patients. Despite this, gluten sensitivity is undertreated and underrecognized as a factor in psychological and neurological disorders [10]. Consequentially, patients with CD who are experiencing extraintestinal symptoms may first see a psychiatrist because these symptoms may reflect psychiatric disorders. Because of this, it is crucial that the psychiatrist be familiar with CD and keep this diagnosis in mind when encountering patients who fit this explanation. Although psychiatric symptoms such as anxiety and depression have been documented, they are rarely discussed in literature reviews. This review focuses on the neurologic and psychiatric manifestations associated with gluten sensitivity, discusses the onset of gluten sensitivity symptoms in celiac disease, and describes the probable processes associated with this pathophysiological response.

## Review

Gluten

Gluten, a protein portion from wheat, rye, barley, and oats, is an essential part of the diet. Globally, gluten sensitivity and the desire for gluten-free products are rising, as everyone eats gluten products which are found mainly as part of each meal. Thus, new, naturally gluten-free baking ingredients and innovative ways to use them are sought [11]. Gluten (from the Latin gluten, meaning "glue") is a combination of prolamins and glutelins, two forms of storage proteins present in cereal (grass) grains. For example, malt is produced from wheat, barley, rye, oats, and hybrids (including spelled Khorasan and emmer, among others). Gluten is what gives dough its elasticity, allowing it to rise and maintain its shape while remaining chewy. Approximately 80% of the protein composition of wheat in bread is gluten. Pasta has a lower gluten content than other grain-based foods. Gluten may also be present in ice cream and ketchup if they contain wheat starch as a stabilizing element. Similarly pervasive is the problem of gluten contamination in other foods. Additionally, hair care and beauty products may contain gluten.

Gluten is crucial for physicians to comprehend because it is associated with a wide range of illnesses, including wheat or grain allergy, dermatitis herpetiformis, gluten ataxia, and celiac disease [12].

Pathophysiology of CD

CD is an autoimmune disorder caused by gluten exposure in genetically predisposed individuals. Chronic lymphocytic inflammation of the small intestine leading to villous atrophy and its related consequences characterize this condition [13]. Those who have a considerable family history of celiac disease (CD) are, in most instances, at the highest risk for developing the condition themselves. The human leukocyte antigen (HLA) molecules HLA-DQ2 and HLA-DQ8 are fundamental components of genetics. Patients who carry both copies of the HLA-DQ2 gene have an extremely elevated risk of developing CD, and HLA-DQ2-positive patients are responsible for more than 90% of all occurrences of CD. These HLA molecules are thought to be responsible for an abnormal adaptive immune response to peptides derived from gluten molecules, and as a result, they play an essential part in the process through which CD develops [14,15].

Prolamine is a storage form for cereal grains, and it is a substance that is both hydrophobic and soluble in alcohol. Gluten is the dietary form of the protein that is responsible for causing an immune response in people with CD. CD4+ T-lymphocytes, which are presented by HLA-DQ2 and HLA-DQ8, are responsible for the majority of the immunological response that is elicited in response to prolamine. A novel avian single-chain fragment variable (scFv) showed positive effects against the peptic-tryptic digest of gliadin (Pt-G), and it may one day be used as a treatment for CD [16].

Non-HLA genetic factors involved in CD immunopathogenesis are unknown. Recent research has revealed the innate immune system's role in autoimmunity. Toll-like receptor (TLR) genetic variants are connected to several autoimmune diseases. TLR expression and activation differ between active CD patients and controls and treated CD patients in several clinical investigations. Due to their potential role in the interaction between the host immune system and some environmental factors (such as viral infections and gut microbiota), TLRs may be included in the list of non-HLA-related genetic factors implicated in CD etiopathogenesis in genetically predisposed individuals exposed to dietary gluten [17]. CD risk is mostly determined by HLA-DQ genes. If the innate and adaptive immune systems fail, the mucosal barrier can be disrupted and localized, or systemic inflammatory and autoimmune processes can occur. Gluten ingestion is the main environmental cause of CD symptoms, although it cannot explain the disease's genesis. To summarize, CD develops from genetic predisposition and gluten consumption. Epidemiological and clinical studies suggest that infections, gut microbiota alterations, and early eating patterns may contribute to illness development. A breakdown in microbiota, innate immunity, genetics, and dietary variables disrupts homeostasis, inflammation, and tissue damage. If we focus on this link and its breakdown, we may be able to understand CD etiology and find novel ways to prevent and cure this prevalent disease [18,19]. Researchers have observed that the amount and quality of gluten eaten, the pattern of infant feeding, and the age at which gluten is introduced to the diet may all influence CD development. Even at two years old, the growing microbiota of children with a genetic predisposition to CD lacks Bacteriodetes, is abundant in Firmicutes, and does not resemble that of adults. Metabolomics may also provide prognostic indicators for CD [20].

Neurological manifestations of CD

CD, a multiorgan disorder with a high rate of extra-intestinal involvement, can cause psychiatric and neurological symptoms, including cerebellar ataxia, peripheral neuropathy, seizures, headache, cognitive impairment, and cortical myoclonus. CD's neurological mechanisms are still debated. Gluten-mediated pathogenesis is associated with antibody cross-reaction, immune-complex deposition, direct neurotoxicity, and extreme vitamin or food deficiency [21]. A gluten-free diet can alleviate most CD symptoms, including neurological manifestations with the exception of cortical myoclonus and dementia, which may require immunosuppressive therapy. Up to this review, there is no consensus on whether serological or neurophysiological data can accurately predict and monitor CD-associated neurological involvement. Moving from the molecular to the symptom level may help us grasp CD's intricate gut-brain relationships. Recognition of multimodal biomarkers may improve "neuroCD" diagnosis, monitoring, and quality of life [21,22].

Treatment should include symptomatic management; however, the hallmark in gluten-related neurological manifestations is embarking on a strict GFD as soon as possible. In the majority of such diseases, with the exemption of cortical myoclonus and advanced dementia, GFD has a positive therapeutic effect. Immunosuppression is only used in cases where strict GFD alone has not been beneficial and in those patients with refractory CD.

Gluten ataxia is a very unusual immune-mediated neurological disease. Antibodies can be found in up to 38% of patients; however, they are usually present at lower titers, making identification difficult [23]. Ataxia's early signs may be subtle, but worsen if left untreated. In a study by Rawat et al. (2022), patients with gluten ataxia were analyzed for structural alterations in different parts of the brain as well as their neurochemical profile in the vermis and right cerebellum. Patients with gluten ataxia had smaller lobes (X) of the cerebellum and thalamus, as well as larger lateral ventricles, compared to healthy controls. Neuronal degeneration was likely present in patients with gluten ataxia, as evidenced by decreased neuronal metabolites and anatomical alterations in the brain [24].

CD increases epilepsy risk, even after diagnosis. Many CD patients have villus atrophy (VA) on follow-up biopsies. A population-based study of CD patients indicated that VA persisting on follow-up biopsy reduced the risk of epilepsy but did not affect hospitalizations for epilepsy emergencies. The mechanism of advantage, the presence of inflammatory mediators, such as tumor necrosis factor (TNF) alpha and neuropeptide Ghrelin, for long-term VA requires further investigation [25]. Epilepsy was more common in children and adolescents who had CD. Patients with unexplained epilepsy should undergo CD screening since early identification and therapy may increase the effectiveness of antiepileptic drugs [26,27].

Celiac patients had a higher prevalence of migraines and tension headaches. There may be a connection between tension headaches and the patient's personality, family, or social environment. Biochemical variables, such as low plasma serotonin, which are present in celiac disease and migraine, may be responsible. Although it is still debatable whether headaches are common in children with celiac disease, strict adherence to a gluten-related diet appears to alleviate neurological symptoms, despite the fact that the underlying pathogenic relationship between celiac disease and headache involvement remains unknown [28].

CD patients often develop peripheral neuropathy and gluten ataxia. A systemic analysis includes 16 papers on CD patients' risk of gluten neuropathy and ataxia. Thirteen studies found gluten neuropathy in CD (up to 39%). Gluten-free diets improve neuropathy and ataxia. In the reported research, gluten neuropathy and gluten ataxia in CD patients differed, but the elevated risk suggests that physicians should evaluate CD in patients with ataxia and neurological symptoms of unknown cause [29].

Most people with CD have some form of mental disability. Memory loss, an inability to calculate, clouded thinking, and personality shifts are typical symptoms. Most CD patients with cognitive impairment have widespread frontal, cortical, subcortical, or thalamic atrophy. Nutrient deficiencies, elevated circulating cytokine levels from systemic inflammation, and low brain serotonin levels have all been hypothesized as reasons for the negative impact of gluten-related diseases on cognitive processes [30]. CD was associated with Alzheimer's and vascular and frontotemporal dementias [31]. Memory, acalculia, inattention, visuospatial, and executive dysfunction should be assessed in CD patients by a neuropsychologist [32].

Psychiatric manifestations of CD

CD is associated with depression, anxiety, eating disorders, autism spectrum disorder (ASD), attention deficit hyperactivity disorder (ADHD), bipolar disorder, schizophrenia, and mood disorders [33]. The relationship between CD and these psychiatric disorders is not well-known or established. Particular biological aspects as well as the effect of a gluten-free diet require additional research.

Depression and Anxiety

The majority of gastrointestinal disorders (GIDs) are associated with depression and anxiety [34]. This suggests that common GID traits, such as prolonged pain and inflammation, influence specific brain targets. The anterior cingulate cortex (ACC) is the primary brain target, which is extremely sensitive to neuroinflammation, and its function explains why GID patients have reduced cognitive and mood status [35]. It was postulated that peripheral neuroinflammation conveys injury or illness to the ACC, which increases threat assessment and pain sensitivity in response to increased vulnerability. Chronic peripheral inflammation overwhelms this process, leading to long-term ACC structural remodeling and elevated threat signals. Even in the absence of clear evidence of threats, this induces anxiodepressive phenotypes because the ACC employs schemas to predict affective outcomes (e.g., pain) based on complex contextual information. This stimulates the autonomic nervous system, aggravates immune dysfunction, and promotes the development of further gastrointestinal illnesses. This theory gives a molecular explanation for how the gastrointestinal, immune, and nervous systems interact in GID, and it is likely that it can also be used to explain other long-term inflammatory diseases [36].

The prevalence of anxiety and depressive symptoms in children was studied using the Revised Children's Anxiety and Depression Scale (RCADS). While 39% of children with celiac disease reported clinically significant concerns for anxiety or depression based on their own RCADS scores (P =0.0001), only 7% of children reported significant concerns for anxiety based on their caregiver-proxy RCADS scores, and 14% reported significant concerns for depression. There was a statistically significant difference between the rates of anxiety and depression symptoms reported by children with and without other medical comorbidities (P = 0.04). Accordingly, it is important to frequently assess pediatric patients with CD for mental health issues, especially anxiety and sadness [37]. Researchers conducted a detailed survey and analysis to discover how often celiacs experience anxiety and depression and how these symptoms are linked to psycho-affective, familial, and lifestyle factors. Celiac patients had 62.7% anxiety and 34.9% depression. Anxiety and sadness were more likely as a result of clinical illnesses and symptoms. Anxiety was caused by a lack of control over CD (98.1%), perceived clinical status (75.0%), daily gluten-free diet problems (63.4%), and daily activities (55.8%). Depression was associated with a lack of CD control (100%), a perceived clinical state (82.2%), and gluten-free diet problems (69.0%) [38]. Following a gluten-free diet religiously may have unintended consequences. Avoiding gluten is essential for people with CD, but some worry that "extreme vigilance" to a gluten-free diet can worsen symptoms like anxiety and fatigue, leading to a diminished quality of life (QOL) [39]. Clinicians must recognize the importance of promoting both dietary adherence and social and emotional well-being in CD patients [40]. It was also speculated that a CD patient's financial situation would have a significant role in shaping their social and emotional concerns. Some people with CD may choose to purchase more GF products than other products as a means of relieving stress due to their low socioeconomic level [39,40].

Having CD has been connected to a lower quality of life and specific mood issues [40]. It is currently unclear how the gluten-free diet may affect these mental elements of the condition. Untreated CD is associated with decreased quality of life and typical complaints of anxiety, melancholy, and exhaustion. Several individuals experience severe psychological morbidity even after their health has improved to some degree after a few months of beginning a gluten-free diet. The quality of life and the ability to stick to a healthy diet may be impacted by psychological disorders. Supporting patients with celiac disease requires that healthcare providers understand the persistent emotional toll the disease has on them [41].

Many studies have shown that persons with CD experience low quality of life, anxiety, and depressive symptoms. They have also highlighted the role that nutrition plays in reducing these effects [38-41]. Yet, very little research has looked into motivation's role and effect on the quality of life and adherence. The outcomes underscore the crucial role that motivation plays in people; in fact, the study revealed a correlation between motivation and diet adherence. So, while treating people with celiac disease, motivation must be taken into consideration [42].

A lack of adherence to a GFD has been linked to an increase in self-reported depressive symptoms, but this link has to be confirmed by longitudinal and prospective studies employing trustworthy measurements, especially for adherence. It's still unclear which comes first, depression or noncompliance [43].

Eating Disorders

Numerous examples of eating disorders (EDs) have been described in celiac disease (CD) patients, implying that ED may be a comorbidity with CD [44]. However, few epidemiological studies have investigated this potential link. While patients with CD were found to have considerably higher Eating Attitude Test scores than controls when testing individuals aged 13 and up, no clear differences were seen between patients with CD and controls when using other screening measures for ED. In order to corroborate these results, further investigations are needed, preferably with larger samples and prospective designs [45]. People with celiac disease may experience disordered eating due to the disease itself, as opposed to gastrointestinal symptoms or psychological issues [46].

Since strict adherence to a GFDis currently the only treatment for celiac disease (CD), there may be other factors that affect how well CD patients adhere to their GFDs, such as food neophobia (FN), which is linked to sensory aversions or fears of the negative effects of eating particular foods. Eating disorders are defined by thoughts (such as the dread of gastrointestinal symptoms after eating and an excessive value placed on one's appearance and weight) and actions (such as dietary restriction and binge eating) linked to physical (such as weight loss) and/or psychological problems (e.g., high distress around eating). It is crucial for gastroenterology clinicians to be aware of past, current, and potential risks for eating disorders given the mounting evidence for a causal link between gastrointestinal illnesses and eating disorders. It is possible that CD may cause FN. This fear, which is more severe in CD patients than in non-CD patients who choose to follow a GFD, can be linked to the possibility of having an unfavorable reaction to gluten-contaminated food products [47,48].

Autism Spectrum Disorder (ASD)

Research in this context indicates a potential genetic link between ASD and CD. However, studies regarding this connection are few and sometimes hampered by small sample sizes and/or a high degree of demographic and clinical variability within ASD groups [49]. The potential for random error and/or systematic bias exists in the vast majority of research that attempts to establish a link between CD and ASD. It is not possible to rule out the possibility of a connection as evidenced by a small but significant number of studies. Larger samples should be recruited for future investigations, and the terms CD and ASD should be defined more precisely [50]. Research into the potential benefits of dietary changes for children with ASD is growing. Though many other sorts of special diets have been advocated as beneficial for people with ASD, gluten has received a lot of attention as a probable aggravating factor. Epidemiological studies have found a link between ASD and CD, and anecdotal evidence shows that a gluten-free diet (GFD) can help improve the behavioral and cognitive issues that ASD patients commonly experience [51]. Because the GFD receives so much public attention, a conversation that focuses specifically on the link between ASD and gluten is essential; yet, both caregivers and physicians have expressed reservations regarding the benefits of gluten-free living for people with ASD [52]. Children on the autism spectrum often exhibit food selectivity (FS), and the dietary consequences of this are well understood. However, the underlying cause of GID in children with ASD caused by changes in gut microbiota is unknown [53]. Despite their frequency, data linking GID to co-occurring disorders have been shown to be inconsistent. It also discovered data that supported the existence of gut-immune-brain linkages by revealing the presence of specific causal interactions. Future research that incorporates large prospective designs and objective and standardized GID measurements will be required to gain a more nuanced knowledge of GID's function in ASD [54].

The etiopathogenesis of ASD comes from a complicated combination of genetic predisposition and environmental variables, affecting people in different ways. Mounting evidence supports the idea that immune system malfunction may play a role in the etiology of ASD for certain people [55]. CD, an autoimmune ailment that mostly affects the small intestine and is driven by gluten consumption, has been linked to ASD by some research while others have shown a random relationship between the two conditions [54,55].

Attention Deficit Hyperactivity Disorder (ADHD)

A putative link between CD and psychiatric and psychological disorders, such as ADHD, has been observed on numerous occasions. CD is significantly overrepresented among ADHD patients. A gluten-free diet greatly improved ADHD symptoms in CD patients [56]. In a search for the incidence of CD among those diagnosed with ADHD, ADHD and control groups had comparable percentages of CD seropositivity. As a result, advocating a gluten-free diet or routine CD testing for children with ADHD makes no sense [57]. The exact co-occurrence of CD and ADHD or the underlying cause of either condition remains unknown. Cognitive problems similar to those seen in children with ADHD, such as a lack of focus or trouble paying attention, were linked to GFD noncompliance in childhood CD, as were psychosomatic symptoms and antisocial behavior [58]. A case of CD that is left untreated increases the likelihood of having symptoms that are comparable to those of ADHD. Due to the presence of ADHD-like cognitive and behavioral features in untreated CD, doctors should be aware of the possibility of elements of ADHD [59].

The debate regarding the relationship between CD and ADHD was addressed by various studies. The majority of experts agree that routine screening for ADHD in people with CD or vice versa should be avoided. At the same time, they hypothesize that individuals with CD and neurological symptoms, including persistent fatigue, inattention, pain, and headache, who are not treated may be at risk for engaging in ADHD-like behavior, specifically inattention (which may be alleviated by following a gluten-free diet). The inattentive subtype of ADHD is phenotypically diverse because it incorporates the clinical construct of slow cognitive tempo (SCT). SCT symptoms are similar to those seen in people with CD who are experiencing neurological complications. Out of 23 studies included in a recent meta-analysis, 13 found a favorable correlation between ADHD and CD [60]. Research demonstrating a favorable correlation has largely appeared in the previous five years. Inconsistencies in the results persist because of the varied methods utilized, notably for ADHD and the outcome questionnaires, as well as a lack of reporting on ADHD subtypes [56,60].

Bipolar Disorder

Bipolar disorders are a complicated category of severe and chronic mental illnesses defined by syndromal manic and hypomanic depressive episodes [61]. In a study by Dickerson F et al. (2011), antibody reactivity to gliadin, deamidated gliadin, and tissue transglutaminase (tTG) was measured and compared between those with and without a history of psychiatric disease. An elevated amount of immunoglobulin G (IgG) antibodies against gliadin has been seen in people with bipolar disorder. On the other hand, the presence of these antibodies is not accompanied by a rise in IgA antibodies to gliadin or the celiac disease-associated antibodies against deamidated gliadin and tTG. These results show that more research is needed into the molecular specificity and pattern of reactivity of the antibody response to gluten antigens in bipolar disorder [62].

CD has very close ties to major depressive disorder, panic disorder, and bipolar disorder. Comorbidity with these disorders is a key predictor of poor quality of life in individuals who have CD. Because of this, it is vital for people who have a CD to report these symptoms to their primary physician to address them earlier in the disease. In addition, it is recommended to perform a screening for CD in individuals who have affective disorders, exhibit essential symptoms, or have a family history of CD [63]. When comorbid with these disorders, the attributable burden of CD in terms of diminishing quality of life was shown to be comparable to that of major chronic diseases, such as Wilson's disease, though it was lower than that of multiple sclerosis when considered alone [64].

Schizophrenia

People with schizophrenia have a two- to fourfold increased risk of dying prematurely compared to the general population. The vulnerability to developing schizophrenia is influenced by both genetic and environmental variables, with drug abuse disorders (especially those involving cannabis) possibly having the strongest link [65]. Past studies have found an association between schizophrenia and CD, but current evidence suggests a causal link has yet to be established [66]. Epidemiological research revealed that those with CD had a noticeably greater risk of developing schizophrenia. Schizophrenia patients face a disproportionately high rate of digestive and liver problems [66,67]. It has been suggested by several studies that removing gluten from the diet of people with schizophrenia can help alleviate some of the symptoms they experience. Although most patients with schizophrenia who had elevated anti-gliadin antibodies (AGA) did not have CD, having elevated antibodies against gliadin is one common immunological abnormality between the two conditions [68]. A treatment-resistant schizophrenia patient with immunological gluten sensitivity benefited from a gluten-restricted diet, showing improvement in both mental and physical symptoms and a reduction in the plasma quantitative level of AGA-IgG [69]. Gluten intolerance is thought to increase chronic inflammation, which, in turn, may worsen the symptoms of schizophrenia and make it more difficult for patients to respond to treatment and absorb medications [65,69].

In the available data, major inconsistencies in the data were found, despite the existing evidence suggesting that patients with celiac disease or gluten allergies may have a slightly higher risk of schizophrenia and mood problems than the general population. GFD is not advised for people with psychosis and mood disorders due to a lack of appropriate research [70].

Other Psychiatric Disorders

Previous research has shown that people with CD are more likely to suffer from neuropsychiatric disorders than the general population. So far, more than 60 non-human leukocyte antigen (HLA) genes have been linked to CD by genome-wide association studies; of these, it is believed that 15% have a role in neurological health [71, 72]. Many common neuropsychiatric disorders include CD as a primary predisposing factor. It's possible that the co-occurrence of diseases is in large part due to shared molecular networks and biological processes. To determine what causes these disorders, we need to look at the underlying molecular mechanisms [72]. CD was associated with an increased risk of psychiatric problems in children, raising their lifetime risk by 1.4 times that of the general population. CD in children has been linked to an increased likelihood of developing psychosocial difficulties later in life, including depression, anxiety, eating disorders, antisocial behavior, attention deficit hyperactivity disorder, autism spectrum disorder, and intellectual disability. It was also more common to have been diagnosed with a mood, eating, or behavioral condition prior to the celiac disease diagnosis. In contrast, no elevated risk was found for any of the psychological diseases studied in the siblings of people with CD [73]. A cohort study included 19,186 children with biopsy-verified celiac disease. Each patient was paired with 5 reference children (controls, n = 94,249). Approximately 16.5% of celiac children were diagnosed with a psychological condition during a median follow-up of 12.3 years, compared to 14.1% of controls [74]. Celiac disease in childhood increased the risk of psychiatric illness by 19% and this risk increases during maturity, in particular, mood, anxiety, eating, ADHD, and autism spectrum problems [75]. There was no statistically significant increase in psychotic disorders, psychoactive substance use, behavioral disorders, personality disorders, suicide attempts, or suicides. CD increases the use of psychiatric medication. Psychological issues associated with CD were also more prevalent [74,75]. As a result, the attending physician should conduct routine surveillance of potential psychiatric symptoms in patients of all ages who have gluten-related diseases, including both children and adults.

Management of CD

The difficulty of treating the symptoms of CD has not decreased despite advances in our knowledge of its genesis, diagnosis, management, and possible medicines for the condition. The understanding that tissue transglutaminase is the autoantigen in CD marked a crucial turning point in the development of the condition. The development of the condition is influenced by environmental factors, but a person's genetic background - including the presence of the HLA-DQ2/DQ8 allele and other non-HLA genes - is crucial (e.g., viral infections and dysbiosis of gut microbiota). The nature of the patient's symptoms will determine which phenotype the doctor will assign them, including but not limited to gastrointestinal, extraintestinal, subclinical, potential, seronegative, non-responsive, or refractory. A small intestine biopsy is still the "gold standard" for diagnosing CD despite the increasing utility of highly sensitive and specific serological assays, including tissue transglutaminase, endomysial, and deamidated gliadin peptide antibodies. Although there is currently no cure for CD, a gluten-free diet can help patients live better, experience fewer symptoms, and have a lower risk of developing complications such as refractory CD, ulcerative jejunoileitis, small intestine adenocarcinoma, and lymphoma. There is still much to learn about several phenotypes of CD such as seronegative, slow-responding, and potential (limited lesions). With the development of substitute and complementary therapies, patients who cannot adhere to a gluten-free diet have cause for optimism [76].

There have been reports of inherent abnormalities between the cells of CD patients and those of healthy individuals, including levels of protein phosphorylation, modifications in vesicular trafficking, and regulation of type 2 transglutaminase (TG2) [77]. Treatments with thapsigargin differentially induced TG2 activation in control and CD cells, as well as endoplasmic reticulum stress activation and autophagic marker expression. Overall, these results showed more molecular characteristics of the celiac cell phenotype and showed that CD cells seemed less able to adapt to stress and respond physiologically [78].

## Conclusions

In conclusion, CD has been linked to several psychiatric manifestations such as depression, anxiety, eating disorders, autism spectrum disorder, attention deficit hyperactivity disorder, bipolar disorder, schizophrenia, and mood disorders. Clinicians should examine mental health factors when a CD diagnosis is established. More research is needed to understand the pathophysiology of CD's psychiatric manifestations.
